# Superfluid qubit systems with ring shaped optical lattices

**DOI:** 10.1038/srep04298

**Published:** 2014-03-06

**Authors:** Luigi Amico, Davit Aghamalyan, Filip Auksztol, Herbert Crepaz, Rainer Dumke, Leong Chuan Kwek

**Affiliations:** 1 CNR-MATIS-IMM & Dipartimento di Fisica e Astronomia, Via S. Sofia 64, 95127 Catania, Italy Centre for Quantum Technologies, National University of Singapore, 3 Science Drive 2, Singapore 117543 and Institute of Advanced Studies, Nanyang Technological University, 1 Nanyang Walk, Singapore 637616; 2 Centre for Quantum Technologies, National University of Singapore, 3 Science Drive 2, Singapore 117543; 3 Centre for Quantum Technologies, National University of Singapore, 3 Science Drive 2, Singapore 117543 and Division of Physics and Applied Physics, Nanyang Technological University, 21 Nanyang Link, Singapore 637371; 4 Centre for Quantum Technologies, National University of Singapore, 3 Science Drive 2, Singapore 117543 and National Institute of Education and Institute of Advanced Studies, Nanyang Technological University, 1 Nanyang Walk, Singapore 637616

## Abstract

We study an experimentally feasible qubit system employing neutral atomic currents. Our system is based on bosonic cold atoms trapped in ring-shaped optical lattice potentials. The lattice makes the system strictly one dimensional and it provides the infrastructure to realize a tunable ring-ring interaction. Our implementation combines the low decoherence rates of neutral cold atoms systems, overcoming single site addressing, with the robustness of topologically protected solid state Josephson flux qubits. Characteristic fluctuations in the magnetic fields affecting Josephson junction based flux qubits are expected to be minimized employing neutral atoms as flux carriers. By breaking the Galilean invariance we demonstrate how atomic currents through the lattice provide an implementation of a qubit. This is realized either by artificially creating a phase slip in a single ring, or by tunnel coupling of two homogeneous ring lattices. The single qubit infrastructure is experimentally investigated with tailored optical potentials. Indeed, we have experimentally realized scaled ring-lattice potentials that could host, in principle, *n* ~ 10 of such ring-qubits, arranged in a stack configuration, along the laser beam propagation axis. An experimentally viable scheme of the two-ring-qubit is discussed, as well. Based on our analysis, we provide protocols to initialize, address, and read-out the qubit.

A qubit is a two state quantum system that can be coherently manipulated, coupled to its neighbours, and measured. Several qubit physical implementations have been proposed in the last decade, all of them presenting specific virtues and bottlenecks at different levels^1^,^2^,^3^,^4^,^5^,^6^. In neutral cold atoms proposals the qubit is encoded into well isolated internal atomic states. This allows long coherence times, precise state readout and, in principle, scalable quantum registers. However, individual qubit (atom) addressing is a delicate point^7^,^8^. Qubits based on Josephson junctions allow fast gate operations and make use of the precision reached by lithography techniques^9^. The decoherence, however, is fast in these systems and it is experimentally challenging to reduce it. For charge qubits the main problem arises from dephasing due to background charges in the substrate; flux qubits are insensitive to the latter decoherence source, but are influenced by magnetic flux fluctuations due to impaired spins proximal to the device^3^.

Here we aim at combining the advantages of cold atom and Josephson junction based implementations. The basic idea is to use the persistent currents flowing through ring shaped optical lattices^10^,^11^,^12^,^14^,^15^ to realize a cold atom analogue of the superconducting flux qubit (see^10^,^16^,^17^,^18^,^19^ for the different schemes that can be applied to induce persistent currents). Recently, superpositions of persistent currents have been thoroughly investigated^14^,^15^.

## Results

In this paper we demonstrate how persistent currents flowing in a ring shaped optical lattice can provide a physical implementation of a qubit^10^,^14^,^15^. The lattice potential plays an important role in our approach. Indeed, it makes strictly one dimensional the atoms' dynamics. Further it provides the means for precise control of the confinement and facilitates the qubit-qubit interaction. In our system we break the Galilean invariance. For a single ring this is realized by creating a localized ‘defect’ barrier along a homogeneous lattice^20^. Additionally we prove that a qubit can be achieved with two homogeneous interacting rings arranged vertically on top of each other. In such a system the Galilean invariance is broken along the direction transverse to the two rings. For this scheme we analyse the real time dynamics and time-of-flight density distributions. Based on our analysis, we provide viable protocols to initialize, address, and read-out the qubits. Indeed, we have experimentally realized scaled ring-lattice potentials that could host, in principle, *n* ~ 10 ring-qubits, arranged in a stack configuration, along the laser beam propagation axis.

### Single-ring-qubit: breaking the Galilean invariance on the single ring with a site defect

**Figure 1:**
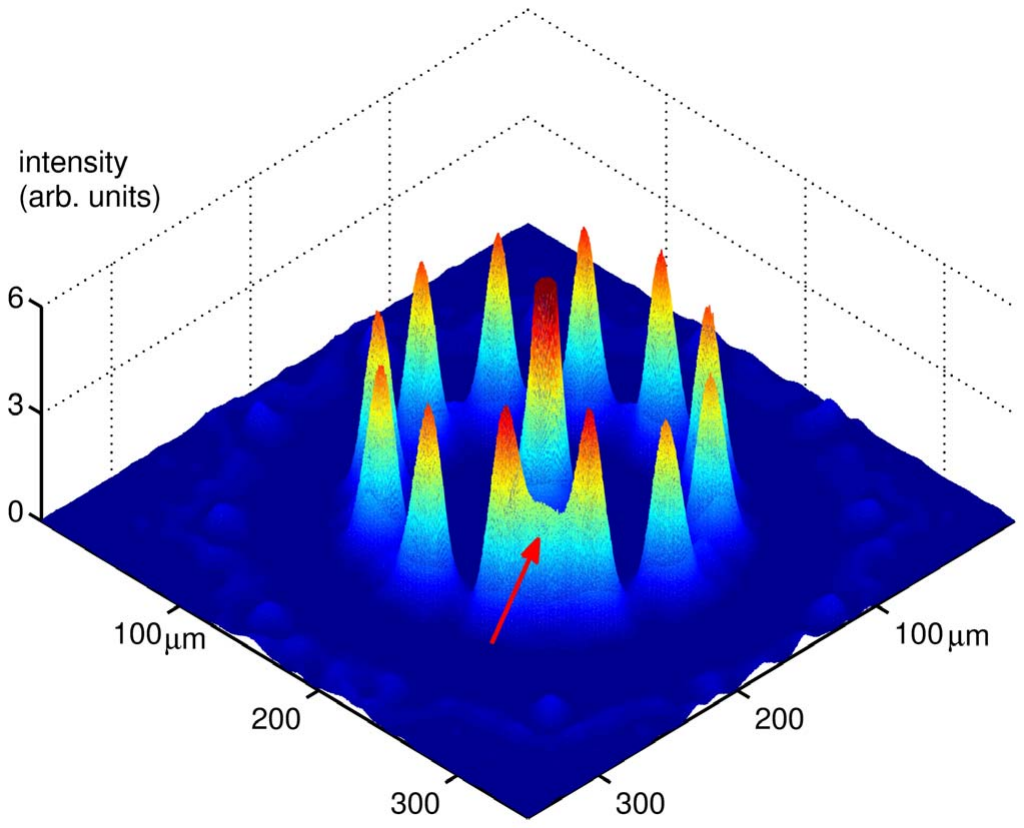
Experimental realization of a ring-lattice potential with an adjustable weak link (red arrow). Measured intensity distribution with an azimuthal lattice spacing of 28 *μ*m and a ring radius of 88 *μ*m (see Methods section). The centre peak is the residual zero-order diffraction. The effective dynamics of a condensate in such a system is governed by the qubit potential as discussed in Eq.(2). The size of the structure is scalable and a lower limit is imposed by the diffraction limit of the focusing optics (see Methods section). Several rings can be arranged in a stack, along the propagation axis of the laser beam (shown in Fig. 5).

We consider bosonic atoms loaded in a ring-shaped potential with identical wells, but with a dimple located at the site *N* − 1 (see Fig. 1), and pierced by a ‘magnetic flux’ Φ. The system is described by the Bose-Hubbard Hamiltonian 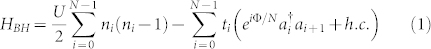
where *a_i_*'s are bosonic operators for atoms trapped in the ring and 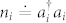. The parameters *t_i_* describe the tunnelling between the wells along the ring. Since the wells are all identical but one, *t_i_* = *t*, µ*i* = 0…*N* − 2 and *t_N_*_ − 1_ = *t*′. Finally, U describes the s-wave scattering interaction^23^. The ‘magnetic flux’ is 
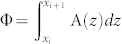
, where ***A****(z)* is the effective vector potential. The effect of the dimple is to induce a phase slip at the site *N* − 1. We assume that the density of superfluid is large enough to neglect the fluctuation of the number of atoms in each well. In this regime we can assume that the system dynamics is characterized by the phases of the superfluid order parameter *ϕ_i_*'s, described by the quantum phase model^24^ with Josephson coupling *J_i_* ~ 〈*n*〉*t_i_* (〈*n*〉 is the average number of bosons in each well). The magnetic flux Φ can be gauged away everywhere but at the site (*N* − 1)-th^25^. Accordingly, the phase difference along nearest neighbour sites can be considered small in the ‘bulk’ and the harmonic approximation can be applied. The partition function can be written as a path integral: 
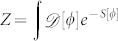
, where the *S*[*ϕ*] is the Euclidean action. Adapting from the approach pursued by Rastelli et al.^29^, all the phases *ϕ_i_* except 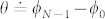 can be integrated out (the integrals are Gaussian). The effective action reads 
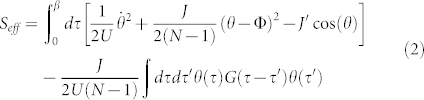
with the potential 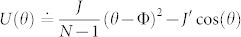. For large (*N* − 1)*J*′/*J* and moderate *N*, *U*(*θ*) defines a two-level system. The degeneracy point is Φ = *π*: The two states are provided by the symmetric and antisymmetric combination of counter-circulating currents corresponding to the two minima of *U*(*θ*). We observe that breaking the Galilean invariance of the system provides an independent parameter *J*′ facilitating the control of the potential landscape. The interaction between *θ* and the (harmonic) bulk degrees of freedom provides the non local term with 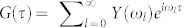, *ω_l_* being Matsubara frequencies and 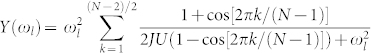. The external bath vanishes in the thermodynamic limit and the effective action reduces to the Caldeira-Leggett one^29^. Finally it is worth noting that the case of a single junction needs a specific approach but it can be demonstrated consistent with Eq.(2).

### Two-rings-qubit: breaking the Galilean invariance with two homogeneous coupled rings

**Figure 2:**
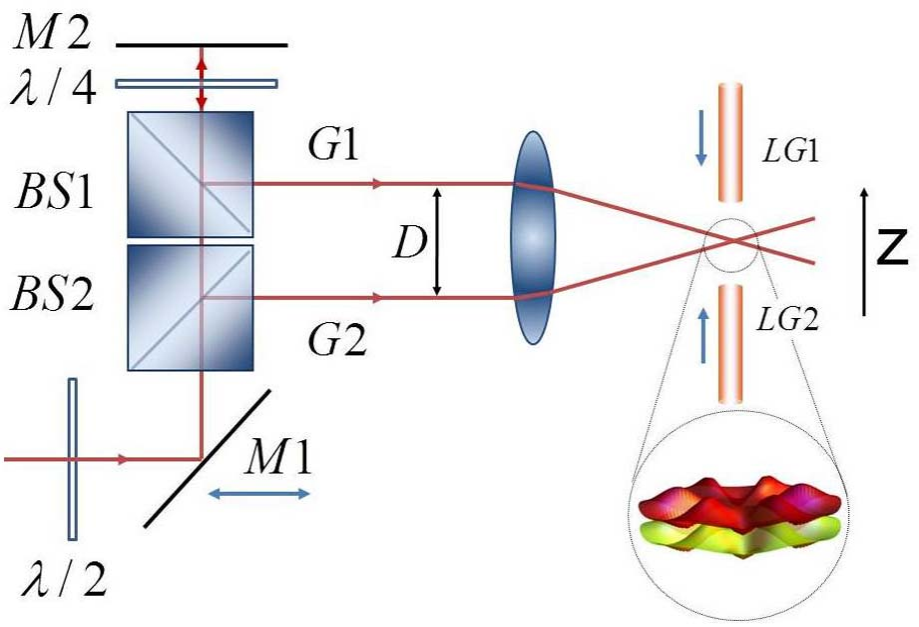
Setup for the ring-ring coupling. Two parallel Gaussian laser beams (G1,G2) are produced by a combination of two polarizing beamsplitter (BS1, BS2). The beam separation *D* can be controlled by moving mirror *M*_1_. Both beams pass through a lens and interfere to form a lattice in z-direction. The distance between the lattice planes is a function of 1/*D*^26^ which can be varied. The resulting one dimensional lattice is combined with vertical beams (LG1, LG2) providing horizontal confinement for trapped atoms (See the Methods section). The inset shows the ring lattice potentials separated by^26^ *d* = *λ*_1_*f*/*D*. The ring-ring separation is adjustable by varying the distance *D*. Such an arrangement provides an effective two-level system that can be exploited as a qubit (See text).

We consider bosonic atoms loaded in two coupled identical *homogeneous* rings Fig. 2. We will prove that such a system effectively provides a qubit-dynamics (alternatively to the one-ring qubit implementation discussed above). The system is described by the Bose-Hubbard ladder: 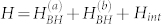, where 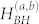 are the Hamiltonians as in Eq.(1) for the bosons in the rings *a* and *b* respectively, and 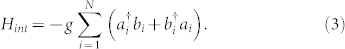
We observe that along each ring the phase slips imply twisted boundary conditions and therefore they can be localized to a specific site, say the *N* − 1-th. Following a similar procedure as employed above, the effective action reads 
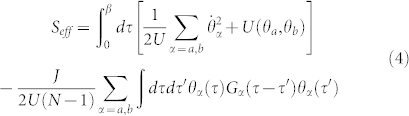
where each *G_α_*(*τ*) is given by the expression found above for the case of a single ring. In this case the phase dynamics is provided by the potential 
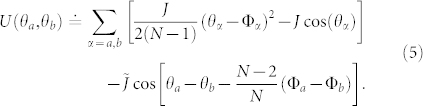
with 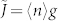
28. We observe that, for large *N*, the potential *U*(*θ_a_*, *θ_b_*) provides that effective phase dynamics of Josephson junctions flux qubits realized by Mooji *et al.* (large *N*'s corresponds to large geometrical inductance of flux qubit devices)^27^. In there, the landscape was thoroughly analysed. The qubit is made with superpositions of the two states |*θ*_1_〉 and |*θ*_2_〉 corresponding to the minima of *U*(*θ_a_*, *θ_b_*). The degeneracy point is achieved by Φ*_b_* − Φ*_a_* = *π*. We comment that the ratio 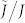 controls the relative size of the energy barriers between minima intra- and minima inter-‘unit cells’ of the (*θ_a_*, *θ_b_*) phase space, and therefore is important for designing the qubit. In our system 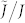 can be fine tuned with the scheme shown in Fig. 2.

Having established that the two tunnel-coupled homogeneous rings, indeed, define a two level system, we now study its real-time dynamics. We will show that the density of the condensate in the two rings can display characteristic oscillations in time.

**Figure 3:**
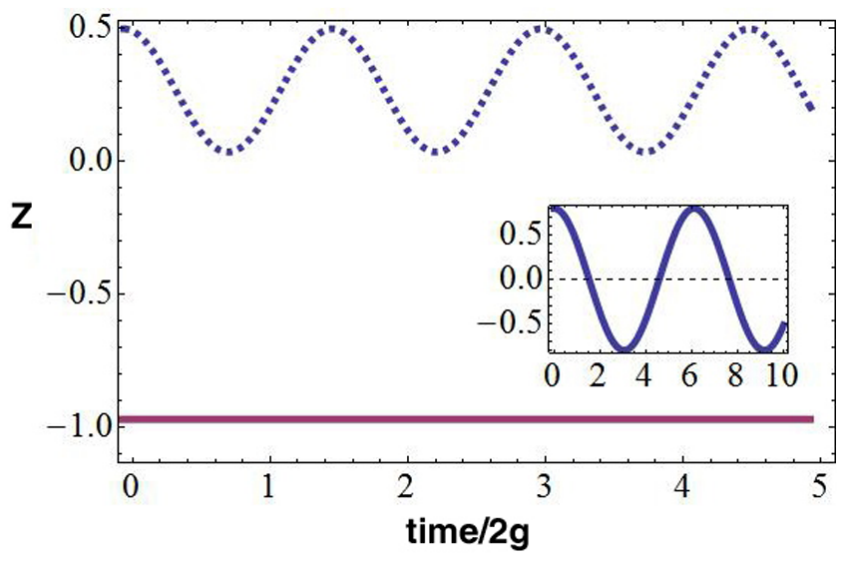
Population imbalance in two coupled rings. We focused on the case 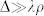. For moderate *z*_0_, oscillations are obtained, with 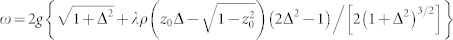 corresponding to macroscopic quantum self trapping (blue dashed line). The dynamics can be visualized with the help of the mechanical system provided by a rotator of length 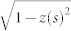, driven by the external force Δ. The constant solution *z*(*s*) = *const* corresponds to vanishing pendulum length (magenta solid line). For Δ = 0 (inset), the dynamics is characterized by Rabi oscillation with 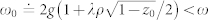. Here *λ_ρ_* = 0.1 and Δ = 4 implying that *ω* ≈ 4*ω*_0_.

**Figure 4:**
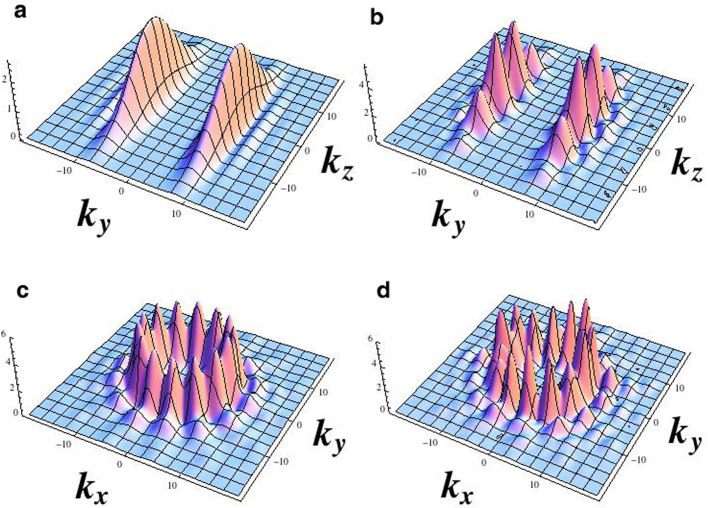
Time-of-flight expansion for the two-coupled-rings-qubit. (a,c), vanishing inter-ring tunnelling rate *g*/*t* = 0. In (b,d), *g*/*t* = 0.9. In the (*k_x_*, *k_y_*) plane the interference fringes with the ring symmetry are due to the momenta of the quantum degenerate gas; the inter-ring tunnelling suppresses the interference fringes. In the (*k_y_*, *k_z_*) plane, *g* induces structured interference fringes. The Eq. (7) is calculated for the Bose-Hubbard ladder with ‘fluxes’ Φ*_a_* and Φ*_b_*, with *U* = 0, and at quantum degeneracy. Results are shown for Φ*_a_* = 80, Φ*_b_* = 70, *T* = 0.05*k_B_* and *N* = 14 with filling fractions of 10 bosons per site.

We make use of the mean field approximation to analyse the (real time) dynamics of the Bose-Hubbard ladder Eqs.(1), (3) (assuming that each ring is in a deep superfluid phase). Accordingly Gross-Pitaevskii equations are found for the quantities depending on the time *s*. *φ_a,i_*(*s*) = 〈*a_i_*(*s*)〉 and *φ_b,i_*(*s*) = 〈*b_i_*(*s*)〉. Assuming that 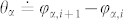 in each ring is site-independent, we obtain 
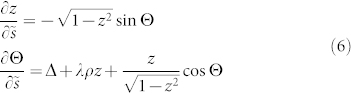
where *z* = (*N_b_* − *N_a_*)/(*N_a_* + *N_b_*) is the normalized imbalance between the populations *N_a_* and *N_b_* of the two rings, Θ = *θ_a_* − *θ_b_* and 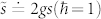. The parameters are 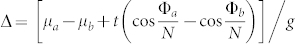, *λ* = *U*/(2*g*), and *ρ* = (*N_a_* + *N_b_*)/*N* is the total bosonic density (we included the chemical potential *μ_α_*). Eqs.(6) can be solved analytically in terms of elliptic functions^28^,^30^. Accordingly, the dynamics displays distinct regimes (oscillating or exponential) as function of the elliptic modulus *k*, depending in turn on Δ, *λ*, and on the initial population imbalance 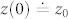. Here we consider the dynamics at 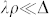, *i.e.* small *U*/*g* (the analysis of the solutions of the Eqs.(6) in different regimes will be presented elsewhere). The results are summarized in Fig. 3. We comment that, comparing with Δ = 0, the oscillations do not average to zero (therefore yielding a macroscopic quantum self trapping phenomenon^30^) and they are faster. The pattern of the circulating currents along the two coupled rings can be read out through the analysis of the time-of-flight density. As customarily, the spatial density distribution in the far field corresponds to the distribution in the momentum space at the time when the confinement potential is turned off: 
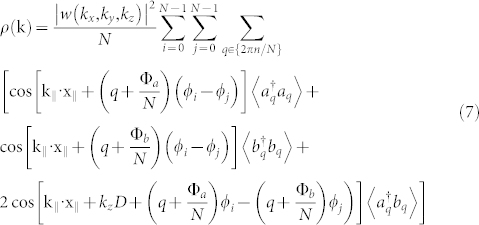
where *w*(*k_x_*,*k_y_*,*k_z_*) are Wannier functions (that we considered identical for the two rings), 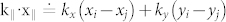, *x_i_* = cos*ϕ_i_*, *y_i_* = sin*ϕ_i_* fix the positions of the ring wells in the three dimensional space, *ϕ_i_* = 2*πi*/*N* being lattice sites along the rings; the expectation values involving the Fourier transforms of operators 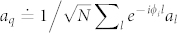 and 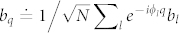 are obtained for *U*/*t* = 0. The density Eq.(7) is displayed in Fig. 4.

## Discussion

We proposed a construction of flux qubits with atomic neutral currents flowing in ring-shaped optical lattice potentials. Persistent currents had been experimentally observed in a narrow toroidal trap with a weak link^31^. The effective action of the system studied in^32^ can provide a two level system. In contrast with^31^,^32^,^33^, we emphasize how we make explicit use of the lattice in our construction, both to confine the particles in the rings and to drive the ring-ring interaction. The qubits are realized by breaking the Galilean invariance of the system either by adding an additional barrier along a single ring lattice Eqs.(2), or by tunnel coupling of two homogeneous rings, Eq.(5). The latter is proposed to be realized with the scheme in Fig. 2. We observe that a suitable variation of such set-up can be exploited also to create two qubit gates (each qubit provided by Fig. 1); alternatively, a route described in the Methods section can be pursued.

The analysis of the real time dynamics of such system can be recast to a type of coupled Gross-Pitaevskii equations that are characteristic for double well potentials, this providing a further proof that the system indeed defines a qubit. Accordingly, the basic phenomenology of the tunnel-coupled homogeneous rings is demonstrated to be characterized by macroscopic quantum self trapping. Since different flow states lead to characteristic density patterns in the far field, standard expansion of the condensate can be exploited to detect the different quantum states of the system (See Fig. 4).

Our work provides a feasible route to the implementation of a functional flux qubit based on persistent atomic currents. For an extensive discussion on the one and two qubit gates, please refer to the Methods section. The initialization of our qubit can be accomplished, for example, imparting rotation by exploiting light induced torque from Laguerre-Gauss (LG) beams carrying optical angular momentum. A two-photon Raman transition between internal atomic states can then be used to transfer coherently 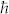 orbital angular momentum to the atoms. With this method, transfer efficiencies of 90% to the rotating state had been demonstrated^31^,^34^. Owing to the coherent nature of the Raman process, superpositions of different angular momentum states can be prepared^37^. Measurements of the decay dynamics of a rotating condensate in an optical ring trap showed remarkable long lifetimes of the quantized flow states on the order of tens of seconds even for high angular momentum (l = 10). Phase slips - the dominant decoherence mechanism - condensate fragmentation and collective excitations which would destroy the topologically protected quantum state are strongly suppressed below a critical flow velocity. Atom loss in the rotating condensate doesn't destroy the state but leads to a slow decrease in the robustness of the superfluid where phase slips become more likely^36^,^39^.

**Figure 5:**
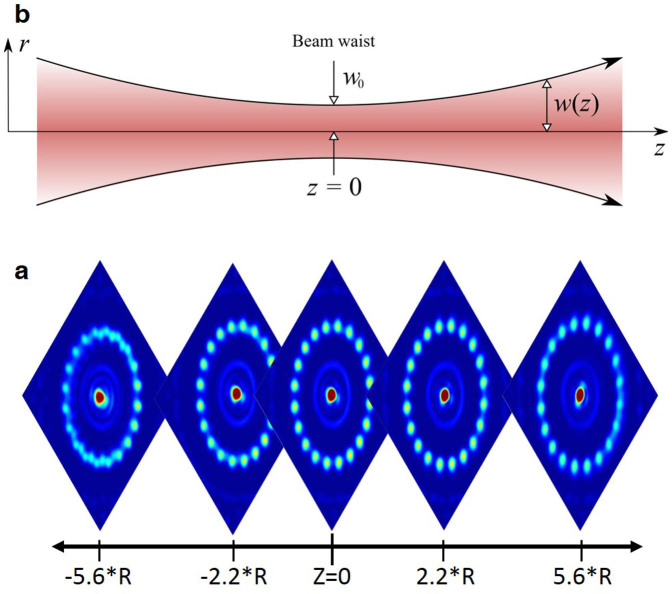
Effect of an axial translation on the ring lattice potential. (a) Ring lattice intensity distribution measured at various positions along the beam propagation axis around the focal plane (Z = 0). Note that the initial beam, phase modified by the SLM, is not Gaussian any more. The optical potential remains undisturbed by a translation of 2.2 times the ring-lattice radius centred around the focal plane (Z = 0). Here R designates the ring-lattice radius of 87.5 *μ*m. (b) This is in contrast to a Gaussian laser beam which exhibits a marked dependence on the axial shift from the focal plane where the beam waist *ω*(*z*) scales with 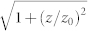 and Rayleigh range *z*_0_.

We comment that, because of the lattice confinement, the gap between the two levels of the qubit displays a favourable scaling with the number of atoms in the system (assuming that the temperature is low enough we can describe the system with Eq.(1))^14^,^15^,^35^. Besides making the inter-ring dynamics strictly one dimensional, the lattice confinement provides the route to the inter-rings coupling. Indeed, the light intensity results to be modulated along the (nearly) cylindrical laser beam. Analysing our experimental configuration, we conclude that it is feasible to arrange *n* ~ 10 ring-qubits in stacks configuration (as sketched in Fig. 5) along the beam propagation axis. To allow controlled tunnelling between neighbouring lattice along the stack, the distance between the ring potentials needs to be adjustable in the optical wavelength regime (the schematics in Fig. 2 can be employed). A trade-off between high tunnelling rates (a necessity for fast gate operations) and an efficient read out and addressability of individual stack sites, needs to be analysed. Increasing the lattice stack separation after the tunnelling interaction has occurred well above the diffraction limit while keeping the atoms confined, optical detection and addressing of individual rings becomes possible.

This arrangement produces equal, adjustable ring-ring spacing between individual vertical lattice sites and can therefore not readily be used to couple two two-ring qubits to perform two-qubit quantum-gates. The SLM method, however, can be extended to produce two ring-lattices in the same horizontal plane, separated by a distance larger than the ring diameter. The separation between these two adjacent rings can then be programmatically adjusted by updating the kinoform to allow tunnelling by mode overlap^46^. Combined with the adjustable vertical lattice (shown in Fig. 2) this would allow, in principle, two-ring qubit stacks to be circumferential tunnel-coupled to form two-qubit gates.

Read out of the angular momentum states can be accomplished experimentally with interference of different flow states (i.e. corresponding to a fragmented superfluid) which maps the phase winding into a density modulation that can be measured using time-of-flight imaging^36^. In the lower panel of Fig. 4 it is shown that different flow states lead to characteristic density patterns in the far field.

We believe that our implementation combines the advantages of neutral cold atoms and solid state Josephson junction based flux qubits for applications in quantum simulation and computation. This promises to exploit the typically low decoherence rates of the cold atom systems, overcoming the single site addressing^40^, and harness the full power of macroscopic quantum phenomena in topologically non trivial systems. The characteristic fluctuations in the magnetic fields affecting Josephson junction based flux qubits are expected to be minimized employing neutral atoms as flux carriers.

## Methods

### Experimental realization of the ring-lattice potential with weak link

We created the optical potential with a liquid crystal on silicon spatial light modulator (LC-R 2500 phase only SLM, Holoeye Photonics AG) which imprints a controlled phase onto a collimated laser beam from a 532 nm wavelength diode pumped solid state (DPSS) laser. The SLM acts as a programmable phase array and modifies locally the phase of an incoming beam. Diffracted light from the computer generated phase hologram then forms the desired intensity pattern in the focal plane of an optical system (doublet lens, f = 150 mm). The resulting intensity distribution is related to the phase distribution of the beam exiting the SLM by Fourier transform. Calculation of the required SLM phase pattern (kinoform) has been carried out using an improved version of the Mixed-Region-Amplitude-Freedom (MRAF) algorithm^20^,^21^ with angular spectrum propagator. This allows us to simulate numerically the wavefront propagation in the optical system without resorting to paraxial approximation. A region outside the desired ring lattice pattern (noise region) is dedicated to collect unwanted light contributions resulting from the MRAF algorithm's iterative optimization process. This can be seen in the measured intensity pattern in Fig. 1 as concentric, periodic structures surrounding the ring-lattice and can be filtered out by an aperture.

The ring-lattice potential shown in Fig. 2 and Fig. 5 can be readily scaled down from a radius of ~90 *μ*m to 5–10 *μ*m by using a 50× microscope objective with NA = 0.42 numerical aperture (Mitutoyo 50× NIR M-Plan APO) as the focusing optics for the SLM beam and with *λ*_2_ = 830 nm light, suitable for trapping Rubidium atoms. Accounting for the limited reflectivity and diffraction efficiency of the SLM, scattering into the noise region and losses in the optical system only about 5% of the laser light contributes to the optical trapping potential. However this is not a limiting factor for small ring-lattice sizes in the tenth of micrometer range as discussed here where ~50 mW laser power is sufficient to produce well depths of several E_rec_. The generated structures are sufficiently smooth, with a measured intensity variation of 4.5% rms, to sustain persistent flow-states^31^. The barrier height can be dynamically modified at a rate up to 50 ms per step, with an upper limit imposed by the frame update rate of the SLM LCD panel (60 Hz).

### Setup for the adjustable ring-ring coupling

To allow controlled tunnelling between neighbouring lattice stacks the distance between the ring potentials needs to be adjustable in the optical wavelength regime. Small distances allow high tunnelling rates, a necessity for fast gate operations. This makes it less efficient to read out and address individual stack sites, however. Increasing the lattice stack separation after the tunnelling interaction has occurred well above the diffraction limit (~*λ*) while keeping the atoms confined, optical detection and addressing of individual rings becomes possible. Fig. 2 in the main text illustrates the experimental arrangement to produce two adjustable 1*d* ring-lattices by intersecting two Gaussian beams (G1,G2) with wavelength *λ*_1_. The inset in Fig. 2 shows two vertically spaced ring lattice potential separated by^26^ *d* = *λ*_1_*f*/*D*. The ring-ring separation is controllable by changing the beam spacing *D* between beams *G*1 and *G*2, allowing adjustment of the ring-ring tunnelling.

In an experimentally feasible arrangement using light from a Ti:Sa laser at *λ*_1_ ≈ 830 nm, with a beam separation adjustable between D = 10–40 mm and a lens focal length *f* = 75 mm, the ring-ring separation can be varied from d = 1.5–6.2 *μ*m. This compares to a inter-ring well spacing of 1.5 *μ*m for a ring lattice with 20 lattice sites and ring radius of 5 *μ*m. Taking advantage of a large ring-ring separation of 5 *μ*m facilitates addressing of individual rings to generate different effective flux-states in a stack. Circulation can be created, for instance, with a pulsed pair of Raman beams where one of the Raman beams carry 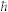 orbital angular momentum. By Raman coupling the |*F* = 2, *m_F_* = 0〉 and |*F* = 2, *m_F_* = 2〉 Zeeman ground-states manifolds of ^87^*Rb* and employing a magnetic gradient field along the vertical axis, the effective two-photon Raman detuning can be shifted out of resonance for atoms in rings other than the addressed one. The differential Zeeman energy shift between the two Raman ground states leads to a magnetic field dependent shift *δ* = *μ_B_g_F_*Δ*m_F_B* of the two-photon Raman detuning. Here *μ_B_* denotes the Bohr magneton, *g_F_* the Landé *g*-factor, Δ*m_F_* the difference between the magnetic spin-quantum numbers of the two Raman states and B the magnetic field strength. With a magnetic field gradient of 180 G/cm – a typical value for magnetic traps in BEC experiments – the two-photon Raman detuning of a ring which is 5 *μ*m separated from the addressed one with *δ* = 0 would be shifted by *δ* = 126 KHz. As was shown by Wright *et al.*^38^, with appropriate choices of the magnitude, intensity ratio and detuning of the Raman beams, fractional population transfer between the |2,2〉 ↔ |2,0〉 states can be accurately controlled by varying the two-photon Raman detuning *δ* in a range of less than 200 KHz. This was demonstrated for Raman beams with Gaussian beam profiles and hence no orbital angular momentum was transferred onto the atoms but it can, in principle, be adapted for a combination of Gaussian and Laguerre-Gaussian beams to generate atomic flux states.

With a SLM arbitrary optical potentials can be produced in a controlled way only in a 2*d*-plane – the focal plane of the Fourier transform lens – making it challenging to extend and up-scale this scheme to 3d trap arrangements. The experiment, however, showed (see Fig. 5) that axially the ring structure potential remains almost undisturbed by a translation along the beam propagation axis of Δz = ±2.2 · R, where *R* denotes the ring-lattice radius. The ring-lattice radius is only weakly affected by an axial shift along z and scales with Δ*R*/*R* = 0.0097 · *z*, where *z* is normalized to the ring-lattice radius. For larger axial shifts from the focal plane the quality of the optical potential diminishes gradually. Based on our measurements this would allow implementation of ring-lattice stacks with more than 10 rings in a vertical arrangement, assuming a stack separation comparable to the spacing between two adjacent lattice sites. Propagation invariant beams may allow a potentially large number of rings to be vertically arranged^44^.

### Tunnelling rate estimation for the two coupled ring lattices

The ring lattice potential shown in the inset in Fig. 2 can be written as 
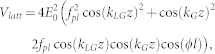
where *f_pl_* are related to Laguerre functions^10^. Such a potential with *l* lattice sites can be created directly by diffraction from a SLM or by superposition of two Laguerre-Gaussian beams with a positive and negative azimuthal index ±*l*, respectively^22^. The WKB estimate of the tunnelling rate gives 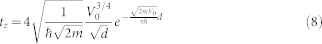
where *d* = *λ f*/*D* is the lattice spacing along z-direction.

### Demonstration of the one qubit and two qubit unitary gates

The aim of this section is to show how the effective phase dynamics of optical ring-lattices with impurities serves the construction of one - and two-qubit gates - a necessity for universal quantum computation. Here, we adapt results which were obtained by Solenov and Mozyrsky^41^ for the case of homogeneous rings with impurities. It results that a single ring optical lattice with impurity is described by the following effective Lagrangian (see Eq. (2) and Supplemental information): 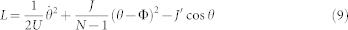
Then we introduce the canonical momentum P in a usual way: 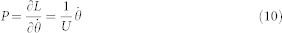
After performing a Legendre transformation we get the following Hamiltonian: 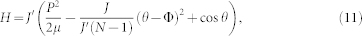
where *μ* = *J*′/*U* is an effective mass of the collective particle. The quantization is performed by the usual transformation *P* → −*d*/*dθ*. For 
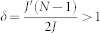
 the effective potential in (11) can be reduced to a double well; for Φ = *π*, the two lowest levels of such double well are symmetric and antisymmetric superpositions of the states in the left and right wells respectively (See the Supplemental material). The effective Hamiltonian can be written as 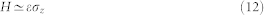
and the lowest two states are |*ψ_g_*〉 = (0,1)*^T^* and |*ψ_e_*〉 = (1,0)*^T^*. An estimate for the gap energy can be found employing the WKB approximation^45^

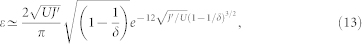
where *δ* > 1. From this formula we can see that the limit of weak barrier and strong interactions is most favourable regime to obtain a finite gap between the two energy levels of the double level potential^12^,^13^,^14^,^35^. We also note that the gap energy splitting can be controlled by the height of the impurity barrier.

### Single qubit gates

For the realization of single-qubit rotations, we consider the system close to the symmetric double well configuration 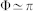. In the basis of the two level system discussed before the Hamiltonian takes the form: 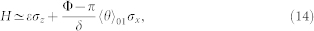
where 〈*θ*〉_01_ is the off-diagonal element of the phase-slip in the two-level system basis. It is easy to show that spin flip, Hadamard and phase gates can be realized by this Hamiltonian. For example, a phase gate can be realized by evolving the state through the unitary transformation *U_z_*(*β*) (tuning the second term of Eq.(14) to zero by adjusting the imprinted flux) 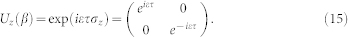
After tuning the gap energy close to zero (adjusting the barrier height of the impurity), we can realize the following rotation 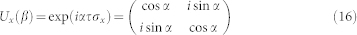
where 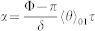. When *α* = *π*/2 and *α* = *π*/4 the NOT and Hadamard gates are respectively realized.

### Two-qubit coupling and gates

The effective dynamics for two coupled qubits, each realized as single ring with localized impurity (as in Fig. 1), is governed by the Lagrangian 
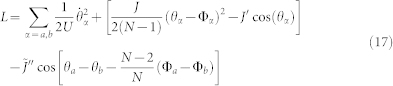
Where *J*″ is the Josephson tunnelling energy between two rings. When Φ*_a_* = Φ*_b_* = Φ and 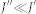 the last term reduces to 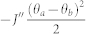 and the Lagrangian takes the form 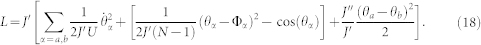
By applying the same procedure as in the previous section, we obtain the following Hamiltonian in the eigen-basis of the two-level systems of rings *a* and *b*

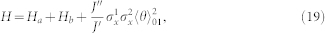

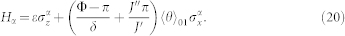
From this equations it follows that qubit-qubit interactions can be realized using our set-up. If we choose the tuning *ε* → 0 and 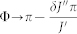 the natural representation of a (*SWAP*)*^α^* gate^42^ can be obtained: 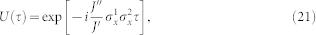
where 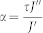. A CNOT gate can be realized by using two 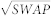 gates. It is well known that one qubit rotations and a CNOT gate are sufficient to implement a set of universal quantum gates^43^.

## Author Contributions

L.A. proposed the idea and developed it further together with D.A. and L.-C.K. H.C. proposed and carried out the experimental implementation with assistance from F.A. and advice from R.D. All authors discussed the results. L.A. and H.C. drafted the manuscript.

## Supplementary Material

Supplementary Information Supplementary Information
