# Supplementary material for: Superfluid qubit systems with ring shaped optical lattices
Source: Sci Rep. 2014 Mar 6;4:4298. doi: 10.1038/srep04298 (PMC3944723; doi:10.1038/srep04298)
Supplement: Supplementary Information [file srep04298-s1.pdf]

Supplemental material for: "Superfluid qubit systems with ring shaped optical lattices"

L. Amico[1,2], D. Aghamalyan[2], H. Crepaz[2], F. Auksztol[2], R. Dumke[2], L.-C. Kwek[2,3]
[1]*CNR-MATIS-IMM & Dipartimento di Fisica e Astronomia, Via S.Sofia 64, 95127 Catania, Italy;* [2]*Centre for Quantum Technologies, National University of Singapore, 3 Science Drive 2, Singapore 117543;* [3]*National Institute of Education and Institute of Advanced Studies, Nanyang Technological University, 1 Nanyang Walk, Singapore 637616*

PACS numbers:

In the Appendix A, the derivation of the effective two-level dynamics of the system (single ring with a dimple) is provided. In Appendix B, we detail on the analysis of the dynamics of phase and population imbalances of coupled persistent currents flowing in the system, respectively. In the Appendix C, details about time-of-flight density distributions plotted in Fig.4 are presented.

Appendix A: Effective qubit dynamics

In this section, we demonstrate how the effective phase dynamics indeed defines a qubit. To this end, we elaborate on the imaginary-time path integral of the partition function of the model Eq.(B1) in the limit of large fluctuations of the number of bosons at each site. We first perform a local gauge transformation $a_l \to a_l e^{il\Phi}$ eliminating the contribution of the magnetic field everywhere except at the weak link site where the phase slip is concentrated[3]). In the regime under scrutiny, the dynamics is governed by the Quantum-Phase Hamiltonian[4]

$$H_{QP} = \sum_{i=0}^{N-2} \left[U n_i^2 - J\cos\left(\phi_{i+1,} - \phi_{i,}\right)\right] + \tag{A1}$$

$$\left[U n_{N-1}^2 - J' \cos\left(\phi_{0,} - \phi_{N-1,} - \Phi\right)\right] \tag{A2}$$

where n_i and ϕ_i are conjugated variables and with $J = t\langle n\rangle$ and $J' = t'\langle n\rangle$.

The partition function of the model Eq.(B1) is

$$Z = Tr\left(e^{-\beta H_{BH}}\right) \propto \int D[\{\phi_i\}] e^{-S[\{\phi_i\}]} \tag{A3}$$

where the effective action is

$$S[\{\phi_i\}] = \int d\tau \sum_{i=0}^{N-2}\left[\frac{1}{U}(\dot{\phi}_i)^2 - J\cos\left(\phi_{i+1,} - \phi_i\right)\right] + \left[\frac{1}{U}(\dot{\phi}_{N-1})^2 - J'\cos\left(\phi_0 - \phi_{N-1} - \Phi\right)\right] \tag{A4}$$

Because of the gauge transformations, the phase slip is produced only at the boundary. We define $\theta \doteq \phi_{N-1,} - \phi_{0,}$. The goal, now, is to integrate out the phase variables in the bulk. To achieve the task, we observe that in the phase-slips-free-sites the phase differences are small, so the harmonic approximation can be applied:

$$\sum_{i=0}^{N-2} \cos\left(\phi_{i+1} - \phi_i\right) \simeq \sum_{i=0}^{N-2} \frac{\left(\phi_{i+1} - \phi_i\right)^2}{2} \;. \tag{A5}$$

In order to facilitate the integration in the bulk phases, we express the single ϕ_0 and ϕ_{N-1} as: $\phi_0 = \tilde{\phi}_0 + \theta/2$, $\phi_{N-1} = \tilde{\phi}_0 - \theta/2$. We observe that the sum of the quadratic terms above involves $N-1$ fields with periodic boundary conditions: $\{\tilde{\phi}_{0,}, \phi_1, \dots, \phi_{N-2}\} \equiv \{\psi_0, \psi_1, \dots, \psi_{N-2}\}$, $\psi_{N-1} = \psi_0$. Therefore

$$\sum_{i=0}^{N-2} \left(\phi_{i+1} - \phi_i\right)^2 = \sum_{i=0}^{N-2} \left(\psi_{i+1} - \psi_i\right)^2 + \frac{1}{2}\theta^2 + \theta\left(\psi_{N-2} - \psi_1\right) \;. \tag{A6}$$

The effective action, $S[\{\phi_i\}]$, can be split into two terms $S[\{\phi_i\}] = S_1[\theta] + S_2[\{\psi_i\}]$ with

$$S_1[\theta] = \int d\tau \left[\frac{1}{U}(\dot{\theta})^2 + \frac{J}{2}\theta^2 - J' \cos(\theta - \Phi) \right] \tag{A7}$$

$$S_2[\{\psi_i\},\theta] = \int d\tau \left\{ \frac{1}{U}(\dot{\psi}_0)^2 + \sum_{i=0}^{N-2} \left[\frac{1}{U}(\dot{\psi}_i)^2 + \frac{J}{2}(\psi_{i+1} - \psi_i)^2 \right] + J\theta(\psi_{N-2} - \psi_1) \right\} \tag{A8}$$

The integration of the fields ψ_i proceeds according to the standard methods (see [5]). The fields that need to be integrated out are expanded in Fourier series (N is assumed to be even): $\psi_l = \psi_0 + (-)^l \psi_{N/2} + \sum_{k=1}^{(N-2)/2} \left(\psi_k e^{\frac{2\pi i k l}{N-1}} + c.c. \right)$, with $\psi_k = a_k + i b_k$. The coupling term in Eq. (A8) involves only the imaginary part of ψ_k: $\psi_{N-2} - \psi_1 = \sum_k b_k \zeta_k$, being $\zeta_k = \frac{4}{\sqrt{N-1}} \sin\left(\frac{2\pi k}{N-1}\right)$. Therefore:

$$S_2[\{\psi_i\},\theta] = \int d\tau \frac{1}{U} \sum_k (\dot{a}_k)^2 + \omega_k^2 a_k^2 + \int d\tau \frac{1}{U} \sum_k (\dot{b}_k)^2 + \omega_k^2 b_k^2 + JU\zeta_k \theta b_k \tag{A9}$$

where $\omega_k = \sqrt{2JU\left[1 - \cos\left(\frac{2\pi k}{N-1}\right)\right]}$. The integral in $\{a_k\}$ leads to a Gaussian path integral; it does not contain the interaction with θ, and therefore brings a prefactor multiplying the effective action, that does not affect the dynamics. The integral in $\{b_k\}$ involves the interaction and therefore leads to a non local kernel in the imaginary time: $\int d\tau d\tau' \theta(\tau) G(\tau - \tau') \theta(\tau')$. The explicit form of $G(\tau - \tau')$ is obtained by expanding $\{b_k\}$ and θ in Matsubara frequencies ω_l. The corresponding Gaussian integral yields to the

$$\int D[b_k] e^{-\int d\tau S_{02}} \propto \exp\left(-\beta U J^2 \sum_{l=0}^{\infty} \tilde{Y}(\omega_l) |\theta_l|^2 \right) \tag{A10}$$

with $\tilde{Y}(\omega_l) = \sum_{k=1}^{(N-2)/2} \frac{\zeta_k^2}{\omega_k^2 + \omega_l^2}$. The $\tau = \tau'$ term is extracted by summing and subtracting $\tilde{Y}(\omega_l = 0)$; this compensates the second term in Eq.(A7).

The effective action finally reads as

$$S_{eff} = \int_0^\beta d\tau \left[\frac{1}{2U}\dot{\theta}^2 + U(\theta) \right] - \frac{J}{2U(N-1)} \sum \int d\tau d\tau' \theta(\tau) G_(\tau - \tau') \theta(\tau') \tag{A11}$$

where

$$U(\theta) \doteq \frac{J}{N-1}(\theta - \Phi)^2 - J' \cos\theta \tag{A12}$$

plotted in Fig.1. The kernel in the non-local term is given by

$$G(\tau) = \sum_{l=0}^{\infty} \sum_{k=1}^{\frac{N-2}{2}} \frac{\omega_l^2 \left(1 + \cos[\frac{2\pi k}{N-1}]\right)}{2JU(1 - \cos[\frac{2\pi k}{N-1}]) + \omega_l^2} e^{i\omega_l \tau} . \tag{A13}$$

The external bath vanishes in the thermodynamic limit and the effective action reduces to the Caldeira-Leggett one [5]. Finally it is worth noting that the case of a single junction needs a specific approach but it can be demonstrated consistent with Eq.(A11).

For the two rings with tunnel coupling, a similar procedure is applied. The effective action (4) is obtained under the assumption that the two rings are weakly coupled and that $U/J << 1$. The effective potential (Eq.(5) of the main manuscript) for the two-rings-qubit is displayed in Fig.2[9].

Appendix B: Real time dynamics: Two coupled Gross-Pitaevskii equations

In this section we study the dynamics of the number and phase imbalance of two bose-condensates confined in the ring shaped potential (see also [9]). A single-species bosonic condensate is envisaged to be loaded in the setup described above. Our system is thus governed by a Bose-Hubbard ladder Hamiltonian

$$H_{BH} = H_a + H_b + H_{int} - \sum_{=a,b} \sum_{i=0}^{N-1} \mu \hat{n}_i \tag{B1}$$

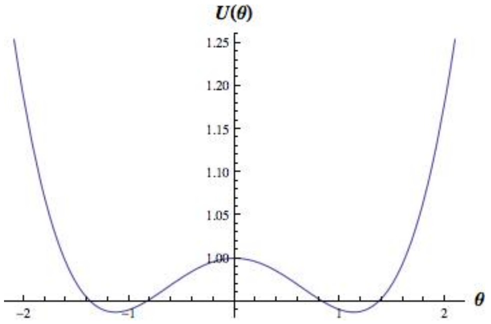

FIG. 1: The double well potential providing the single-ring-qubit for $J/[J'(N-1)] = 0.4$ and $\Phi = \pi$

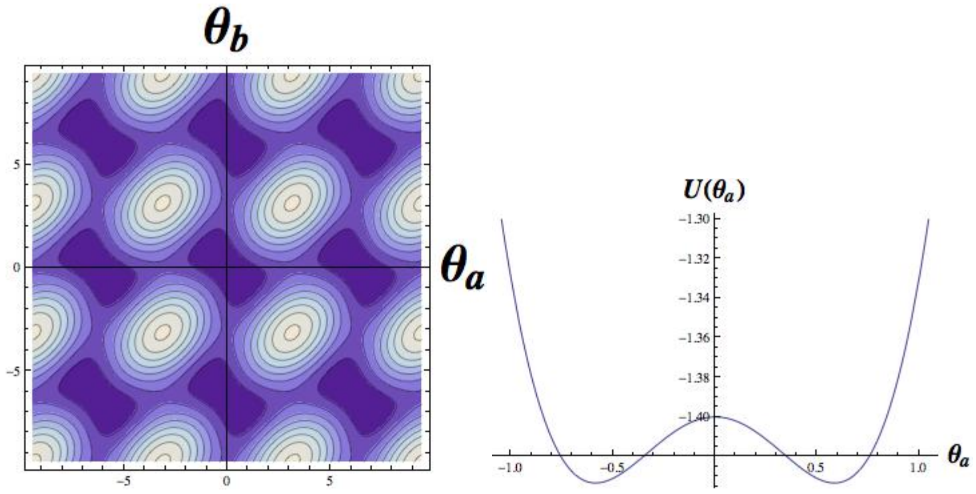

FIG. 2: (Left) The effective potential landscape providing the two-rings-qubit. (Right) The double well for $\theta_a = -\theta_b$. The parameters are $\tilde{J}/J = 0.8$ and $\Phi_a - \Phi_b = \pi$.

with

$$H_a = -t\sum_{i=0}^{N-1}(e^{i\Phi_a/N}a_i^\dagger a_{i+1} + h.c.) + \frac{U}{2}\sum_{i=1}^{N}\hat{n}_i^a(\hat{n}_i^a - 1)$$
$$H_b = -t\sum_{i=0}^{N-1}(e^{i\Phi_b/N}b_i^+ b_{i+1} + h.c.) + \frac{U}{2}\sum_{i=1}^{N}\hat{n}_i^b(\hat{n}_i^b - 1)$$
$$H_{int} = -g\sum_{i=0}^{N-1}(a_i^\dagger b_i + b_i^\dagger a_i) \quad \text{(B2)}$$

where $H_{a,b}$ are the Hamiltonians of the condensates in the rings a and b and the H_{int} describes the interaction between rings. Operators $\hat{n}_i^a = a_i^\dagger a_i, \hat{n}_i^b = b_i^\dagger b_i$ are the particle number operators for the lattice site i. Operators a_i and b_i obey the standard bosonic commutation relations. The parameter t is the tunneling rate within lattice neighboring sites, and g is the tunneling rate between the rings. The on-site repulsion between two atoms is quantified by $U = \frac{4\pi a_s \hbar^2}{m}\int |w(\mathbf{x})|^4 d^3\mathbf{x}$, where a_s is the s-wave scattering length of the atom and $|w(\mathbf{x})|$ is a single-particle Wannier function. Finally, the phases Φ_a and Φ_b are the phase twists responsible for the currents flowing along the rings. They can be expressed through vector potential of the so-called synthetic gauge fields in the following way: $\Phi_a/N = \int_{x_i}^{x_{i+1}} \boldsymbol{A(z)}dz$, $\Phi_b/N = \int_{x_i}^{x_{i+1}} \boldsymbol{B(z)}dz$, where $\boldsymbol{A(z)}$ and $\boldsymbol{B(z)}$ are generated vector potentials in the rings a and b, respectively. We would like to emphasize, that the inter-ring hopping element g is not affected by the Peierls substitution because the synthetic gauge field is assumed to have components longitudinal to the rings only.

To obtain the Gross-Pitaevskyi, we assume that the system is described by a Bose-Hubbard ladder Eqs.(B1), is in a superfluid regime, with negligible quantum fluctuations. The order parameters can be defined as the expectation values of bosonic operators in the Heisenberg picture:

$$\varphi_{a,i}(s) = \langle a_i(s)\rangle, \varphi_{b,i}(s) = \langle b_i(s)\rangle\ , \quad \text{(B3)}$$

implying that the Heisenberg equations for the operators a_i and b_i are simplified into the Gross-Pitaevskii equations for the corresponding expectation values:

$$i\hbar\frac{\partial\varphi_{a,i}}{\partial s} = -t(e^{i\Phi_a/N}\varphi_{a,i+1} + e^{-i\Phi_a/N}\varphi_{a,i-1}) \\ +U|\varphi_{a,i}|^2\varphi_{a,i} - \mu_a\varphi_{a,i} - g\varphi_{b,i} \tag{B4}$$

$$i\hbar\frac{\partial\varphi_{b,i}}{\partial s} = -t(e^{i\Phi_b/N}\varphi_{b,i+1} + e^{-i\Phi_b/N}\varphi_{b,i-1}) \\ +U|\varphi_{b,i}|^2\varphi_{b,i} - \mu_b\varphi_{b,i} - g\varphi_{a,i} \tag{B5}$$

We assume that $\varphi_{a,i+1} - \varphi_{a,i} = \frac{\varphi_a(s)}{\sqrt{N}}$ and $\varphi_{b,i+1} - \varphi_{b,i} = \frac{\varphi_b(s)}{\sqrt{N}}$ for all $i, j = 0, .., N$, where N is a total number of ring-lattice sites. From Eqs.(B4) and (B5) we obtain

$$i\hbar\frac{\partial\varphi_a}{\partial s} = -2t\cos(\Phi_a/N)\varphi_a + \frac{U}{N}|\varphi_a|^2\varphi_a \\ -\mu_a\varphi_a - g\varphi_b \tag{B6}$$

$$i\hbar\frac{\partial\varphi_b}{\partial s} = -2t\cos(\Phi_b/N)\varphi_b + \frac{U}{N}|\varphi_b|^2\varphi_b \\ -\mu_b\varphi_b - g\varphi_a \tag{B7}$$

Employing the standard phase-number representation: $\varphi_{a,b} = \sqrt{N_{a,b}}e^{i\theta a,b}$, two pairs of equations are obtained for imaginary and real parts:

$$\hbar\frac{\partial N_a}{\partial s} = -2g\sqrt{N_a N_b}\sin(\theta b - \theta a)$$

$$\hbar\frac{\partial N_b}{\partial s} = 2g\sqrt{N_a N_b}\sin(\theta b - \theta a) \tag{B8}$$

$$\hbar\frac{\partial\theta a}{\partial s} = -2t\cos\Phi_a/N - \frac{UN_a}{N} + \mu_a + g\sqrt{\frac{N_b}{N_a}}\cos(\theta b - \theta a)$$
$$\hbar\frac{\partial\theta b}{\partial s} = -2t\cos\Phi_b/N - \frac{UN_b}{N} + \mu_b + g\sqrt{\frac{N_a}{N_b}}\cos(\theta b - \theta a) \tag{B9}$$

From Eqs.(B8) it results that $\frac{\partial N_a}{\partial s} + \frac{\partial N_b}{\partial s} = 0$, reflecting the conservation of the total bosonic number $N_T = N_a + N_b$. From equations (B8) and (B9) we get

$$\frac{\partial z}{\partial\tilde{s}} = -\sqrt{1 - z^2}\sin\Theta \tag{B10}$$

$$\frac{\partial\Theta}{\partial\tilde{s}} = \Delta + \lambda\rho z + \frac{z}{\sqrt{1 - z^2}}\cos\Theta \tag{B11}$$

where we introduced new variables:the dimensionless time $2gs/\hbar \to \tilde{s}$,the population imbalance $z(\tilde{s}) = (N_b - N_a)/(N_a + N_b)$ and the phase difference between the two condensates $\Theta(\tilde{s}) = \theta a - \theta b$. It is convenient to characterize the system with a new set of parameters: external driving force $\Delta = (2t(\cos\Phi_a/N - \cos\Phi_b/N) + \mu_b - \mu_a)/2g$, effective scattering wavelength $\lambda = U/2g$ and total bosonic density $\rho = N_T/N$. The exact solutions of Eqs.(B10) and (B11) in terms of elliptic functions[10] can be adapted to our case[9]. The equations can be derived as Hamilton equations with

$$H(z(\tilde{s}), \Theta(\tilde{s})) = \frac{\lambda\rho z^2}{2} + \Delta z - \sqrt{1 - z^2}cos\Theta, \tag{B12}$$

by considering z and ϕ as conjugate variables. Since the energy of the system is conserved, $H(z(\tilde{s}),\Theta(\tilde{s})) = H(z(0),\Theta(0)) = H_0$. Combining Eqs.(B10) and (B12), Θ can be eliminated, obtaining

$$\dot{z}^2 + [\frac{\lambda\rho z^2}{2} + \Delta z - H_0]^2 = 1 - z^2, \tag{B13}$$

that is solved by quadratures:

$$\frac{\lambda\varrho\tilde{s}}{2} = \int_{z(0)}^{z(\tilde{s})} \frac{dz}{\sqrt{f(z)}}\,, \tag{B14}$$

where $f(z)$ is the following quartic equation

$$f(z) = \big(\frac{2}{\lambda\rho}\big)^2(1-z^2) - \big[z^2 + \frac{2z\Delta}{\lambda\rho} - \frac{2H_0}{\lambda\rho}\big]^2\,. \tag{B15}$$

There are two different cases: $\Delta = 0$ and $\Delta \neq 0$.

I) $\Delta = 0$. – In this case the solution for the $z(t)$ can be expressed in terms of 'cn' and 'dn' Jacobian elliptic functions as([10]):

$$\begin{aligned} z(\tilde{s}) &= Ccn[(C\lambda\rho/k(\tilde{s}-\tilde{s}_0),k)] \;\; for \;\; 0<k<1 \\ &= Csech(C\lambda\rho(\tilde{s}-\tilde{s}_0)), \;\; for \;\; k=1 \\ &= Cdn[(C\lambda\rho/k(\tilde{s}-\tilde{s}_0),1/k)] \;\; for \;\; k>1; \qquad \text{(B16)} \\ k &= \big(\frac{C\lambda\rho}{\sqrt{2}\zeta(\lambda\rho)}\big)^2 = \frac{1}{2}\big[1 + \frac{(H_0\lambda\rho - 1)}{(\lambda\rho)^2 + 1 - 2H_0\lambda\rho}\big], \qquad \text{(B17)} \end{aligned}$$

where

$$\begin{aligned} C^2 &= \frac{2}{(\lambda\rho)^2}((H_0\lambda\rho-1)+\zeta^2), \\ {}^2 &= \frac{2}{(\lambda\rho)^2}(\zeta^2 - (H_0\lambda\rho - 1)), \\ \zeta^2(\lambda\rho) &= 2\sqrt{(\lambda\rho)^2 + 1 - 2H_0\lambda\rho}, \end{aligned} \tag{B18}$$

and $\tilde{s}_0$ fixing $z(0)$. Jacobi functions are defined in terms of the incomplete elliptic integral of the first kind $F(\phi,k) = \int_0^\phi d\theta(1-k\sin^2\theta)^{-1/2}$ by the following expressions: $sn(u|k) = \sin\phi, cn(u|k) = \cos\phi$ and $dn(u|k) = (1-k\sin^2\phi)^{1/2}$ [12]. The Jacobian elliptic functions $sn(u|k)$, $cn(u|k)$ and $dn(u|k)$ are periodic in the argument u with period $4K(k)$, $4K(k)$ and $2K(k)$, respectively, where $K(k) = F(\pi/2,k)$ is the complete elliptic integral of the first kind. For small elliptic modulus $k \simeq 0$, such functions behave as trigonometric functions; for $k \simeq 1$, they behave as hyperbolic functions. Accordingly, the character of the solution of Eqs.(B10) and (B11) can be oscillatory or exponential, depending on k. For $k \ll 1$, $cn(u|k) \approx \cos u + 0.25k(u - \sin(2u)/2)\sin u$ is almost sinusoidal and the population imbalance is oscillating around a zero average value. When k increases, the oscillations become non-sinusoidal and for $1-k \ll 1$ the time evolution is non-periodic: $cn(u|k) \approx \sec u - 0.25(1-k)(\sinh(2u)/2 - u)\tanh u \sec u$. From the last expression, we can see that at $k = 1$, $cn(u|k) = \sec u$ so oscillations are exponentially suppressed and $z(\tilde{s})$ taking 0 asymptotic value. For the values of the $k>1$ such that $[1-1/k] \ll 1$ $z(s)$ is still non-periodic and is given by: $dn(u|1/k) \approx \sec u + 0.25(1-1/k)(\sinh(2u)/2 + u)\tanh u \sec u$. Finally when $k \gg 1$ than the behavior switches to sinusoidal again, but $z(\tilde{s})$ does oscillates around a non-zero average: $dn(u|1/k) \approx 1 - \sin^2 u/2k$. This phenomenon accounts for the MQST.

II)$\Delta \neq 0$.– In this case $z(s)$ is expressed in terms of the Weierstrass elliptic function([10, 11])

$$z(\tilde{s}) = z_1 + \frac{f'(z_1)/4}{\wp(\frac{\lambda\rho}{2}(\tilde{s}-\tilde{s}_0); g_2, g_3) - \frac{f''(z_1)}{24}}\,, \tag{B19}$$

where $f(z)$ is given by an expression (B15), z_1 is a root of quartic $f(z)$ and $\tilde{s}_0 = (2/\lambda\rho)\int_{z_1}^{z(0)} \frac{dz'}{\sqrt{f(z')}}$. For $\sin\Theta_0 = 0$ (which is the case discussed in the text), $z_1 = z_0$ and consequently $s_0 = 0$. The Weierstrass elliptic function can be given as the inverse of an elliptic integral $\wp(u; g_2, g_3) = y$, where

$$u = \int_y^\infty \frac{ds}{\sqrt{4s^3 - g_2 s - g_3}}\,. \tag{B20}$$

The constants g_2 and g_3 are the characteristic invariants of ϱ:

$$\begin{aligned} g_2 &= -a_4 - 4a_1a_3 + 3a_2^2 \\ g_3 &= -a_2a_4 + 2a_1a_2a_3 - a_2^3 + a_3^2 - a_1^2a_4, \end{aligned} \tag{B21}$$

where the coefficients a_i, where $i = 1, .., 4$, are given as

$$\begin{aligned} a_1 &= -\frac{\Delta}{\lambda\rho}; a_2 = \frac{2}{3(\lambda\rho)^2}(\lambda\rho H_0 - (\Delta^2+1)) \\ a_3 &= \frac{2H_0\Delta}{(\lambda\rho)^2}; a_4 = \frac{4(1-H_0^2)}{(\lambda\rho)^2} \end{aligned} \tag{B22}$$

In the present case ($\Delta \neq 0$), the discriminant

$$\delta = g_2^3 - 27g_3^2 \tag{B23}$$

of the cubic $h(y) = 4y^3 - g_2y - g_3$ governs the behavior of the Weierstrass elliptic functions (we contrast with the case $\Delta = 0$, where the dynamics is governed by the elliptic modulus k). If $g_2 < 0, g_3 > 0$ then([12])

$$z(\tilde{s}) = z_1 + \frac{f'(z_1)/4}{c + 3c\sinh^{-2}[\frac{\sqrt{3c}\lambda\rho}{2}(\tilde{s}-\tilde{s}_0)] - \frac{f''(z_1)}{24}} . \tag{B24}$$

Namely, the oscillations of z are exponentially suppressed and the population imbalance decay (if $z_0 > 0$) or saturate (if $z_0 < 0$) to the asymptotic value given by $z(\tilde{s}) = z_1 + \frac{f'(z_1)/4}{c - f''(z_1)/24}$.
If $g_2 > 0, g_3 > 0$ then([12])

$$z(\tilde{s}) = z_1 + \frac{f'(z_1)/4}{-c + 3c\sin^{-2}[\frac{\sqrt{3c}\lambda\rho}{2}(\tilde{s}-\tilde{s}_0)] - \frac{f''(z_1)}{24}} , \tag{B25}$$

where $c = \sqrt{g_2/12}$. We see that the population imbalance oscillates around a non-zero average value $\overline{z} \doteq z_1 + \frac{f'(z_1)/4}{2(2c - f''(z_1)/24)}$, with frequency $\omega = 2g\sqrt{3c}\lambda\rho$.

We express the Weierstrass function in terms of Jacobian elliptic functions. This leads to significant simplification for the analysis of these regimes.

For $\delta > 0$, it results

$$z(\tilde{s}) = z_1 + \frac{f'(z_1)/4}{e_3 + \frac{e_1 - e_3}{sn^2[\frac{\lambda\rho\sqrt{e_1-e_3}}{2}(\tilde{s}-\tilde{s}_0), k_1]} - \frac{f''(z_1)}{24}} , \tag{B26}$$

where $k_1 = \frac{e_2 - e_3}{e_1 - e_3}$ and e_i are solutions of the cubic equation $h(y) = 0$. In this case the population imbalance oscillates about the average value $\overline{z} = z_1 + \frac{f'(z_1)/4}{2(e_1 - f''(z_1)/24)}$.

The asymptotics of the solution is extracted through: $k \ll 1$, $sn(u|k) \approx \sin u - 0.25k(u - \sin(2u)/2)\cos u$. When k increases oscillations starting to become non-sinusoidal and when $1 - k \ll 1$ it becomes non-periodic and takes form: $cn(u|k) \approx \tanh u - 0.25(1-k)(\sinh(2u)/2 - u)\sec^2 u$.

For $\delta < 0$ the following expression for $z(s)$ is obtained:

$$z(\tilde{s}) = z_1 + \frac{f'(z_1)/4}{e_2 + H_2\frac{1 + cn[\lambda\rho\sqrt{H_2}(\tilde{s}-\tilde{s}_0), k_2]}{1 - cn[\lambda\rho\sqrt{H_2}(\tilde{s}-\tilde{s}_0), k_2]} - \frac{f''(z_1)}{24}} , \tag{B27}$$

where $k_2 = 1/2 - \frac{3e_2}{4H_2}$ and $H_2 = \sqrt{3e_2^2 - \frac{g_2}{4}}$.The asymptotical behavior of the function $cn(u|k)$ has been discussed in the previous subsection. As it it seen from this expression $z(\tilde{s})$ oscillates about the average value $\overline{z} = z_1 + \frac{f'(z_1)/4}{2(e_2 - f''(z_1)/24)}$.

1. Population imbalance and oscillation frequencies in the limit $\lambda\rho \ll 1$

I-B $\Delta = 0$.– The qualitative behavior of the dynamics for this sub-case depends on the elliptic modulus k which is given by Eq.(B17). For $\lambda\rho \ll 1$

$$k = z(0)\lambda\rho(1 - \frac{\lambda\rho}{2}\sqrt{1 - z(0)^2}) \tag{B28}$$

implying that $k \approx 0$; therefore $z(t)$ displays only one regime given by

$$z(\tilde{s}) \simeq z(0)(\cos\omega(\tilde{s}-\tilde{s}_0) + \frac{k}{4}(\omega(\tilde{s}-\tilde{s}_0) - \sin 2\omega(\tilde{s}-\tilde{s}_0))\sin\omega(\tilde{s}-\tilde{s}_0)) . \tag{B29}$$

where $\omega \simeq 2g(1+\frac{\lambda}{2}\rho\sqrt{1-z(0)^2})$ and $\tilde{s}_0$ is fixing initial condition. Therefore, in this regime the population imbalance is characterized by almost sinusoidal oscillations about zero average– see the inset of Fig. 3 of the main part of the material.

II-B $\Delta \neq 0$.– In this case, the behavior of $z(t)$ is governed by the discriminant δ of the cubic equation Eq.(B23). There are two different regimes depending on the initial value of the population imbalance which are given by the value of δ. All the regimes can be discussed by expressing the Weierstrass function in Eq.(B19) using Jacobian elliptic functions. In the limit of $\delta = 0$, the population imbalance is

$$z(\tilde{s}) = z(0) + \frac{f'[z(0)]/4}{-c + 3c[\sin(-\sqrt{3c}\frac{\lambda\rho}{2}\tilde{s})]^{-2} - f''[z(0)]/24} . \tag{B30}$$

For the parameters discussed in Fig. 3 of the main article, $f'[z(0)] \sim 10^{-14}$; therefore the population imbalance is constant due to the same reason discussed for $D = 0$ above. In the limit of $\delta < 0$, the population imbalance is

$$z(\tilde{s}) = z(0) + \frac{f'[z(0)]/4}{e_2 + H_2\frac{1+\cos(\lambda\rho\sqrt{H_2}\tilde{s})}{1-\cos(\lambda\rho\sqrt{H_2}\tilde{s})} - f''[z(0)]/24} , \tag{B31}$$

where $e2, H_2$ are defined in the Appendix A. Eq.(B31) is correct when $1/2 - 3e_2/4H_2 \simeq 0$(for the parameters considered in the article $m \simeq 10^{-7}$). As one sees from this formula, the population imbalance displays an oscillating behavior around a non-zero average (MQST regime) with frequency given by

$$\omega = 2g(\sqrt{1+\Delta^2} + \frac{(z(0)\Delta - \sqrt{1-z(0)^2})(2\Delta^2-1)}{2(1+\Delta^2)^{3/2}}\lambda\rho) . \tag{B32}$$

This two regimes are shown in Fig. 3 of the main article.

Appendix C: Time of flight

In this section the density of momentum distribution which can be observed in the time of flight type of measurement for a Bose-Hubbard ladder model Eq.(B1) is derived. The density of momentum distribution is given by

$$\rho(\mathbf{k}) = \int d^3x \int d^3x' \langle\Psi(\mathbf{x})^\dagger\Psi(\mathbf{x}')\rangle e^{i\mathbf{k}(\mathbf{x}-\mathbf{x}')} , \tag{C1}$$

where $\Psi^\dagger(\mathbf{x})$ and $\Psi(\mathbf{x}')$ are bosonic field-operators.

Let us express them through Wannier functions:

$$\Psi(\mathbf{x}) = \sum_{i=0}^{N-1}[w(\mathbf{x}-\mathbf{r}_i)e^{i\varphi_i^a}a_i + w(\mathbf{x}-\mathbf{r}_i)e^{i\varphi_i^b}b_i] , \tag{C2}$$

where exponential factors arise from the Peierls substitution and they are given by $\varphi_{i+1}^a - \varphi_i^a = 2\pi\Phi_a/L^2$ and $\varphi_{i+1}^b - \varphi_i^b = 2\pi\Phi_b/L^2$, where Φ_a and Φ_b are the fluxes induced in the rings a and b respectively. After substituting Eq.(C2) into Eq.(C1) and making change of variables $\mathbf{z} = \mathbf{x} - \mathbf{r}_i$,$\mathbf{z}' = \mathbf{x}' - \mathbf{r}_i$, we get

$$\rho(\mathbf{k}) = \sum_i\sum_j[|w(\mathbf{k})|^2(e^{i(\varphi_j^a-\varphi_i^a)}\langle a_i^\dagger a_j\rangle + e^{i(\varphi_j^b-\varphi_i^b)}\langle b_i^\dagger b_j\rangle + e^{i(\varphi_j^a-\varphi_i^b)}\langle b_i^\dagger a_j\rangle + e^{i(\varphi_j^b-\varphi_i^a)}\langle a_i^\dagger b_j\rangle)]e^{i\mathbf{k}(\mathbf{r}_i-\mathbf{r}_j)} . \tag{C3}$$

We note that $z_i - z_j = 0$ for i and j belonging to the same ring; otherwise $z_i - z_j = \pm D$, D being the distance between the rings.Therefore, the momentum distribution reads

$$
\begin{aligned}
\rho(\mathbf{k}) &= |w(\mathbf{k})|^2[\sum_{i\in a}\sum_{j\in a}(e^{i((\varphi^a_j-\varphi^a_i)+\mathbf{k}_\parallel\cdot\mathbf{x}_\parallel)}\langle a^\dagger_i a_j\rangle \\
&+ \sum_{i\in b}\sum_{j\in b} e^{i((\varphi^b_j-\varphi^b_i)+\mathbf{k}_\parallel\cdot\mathbf{x}_\parallel)}\langle b^\dagger_i b_j\rangle \\
&+ \sum_{i\in a}\sum_{j\in b} e^{i((\varphi^b_j-\varphi^a_i)+\mathbf{k}_\parallel\cdot\mathbf{x}_\parallel+k_z D)}\langle b^\dagger_i a_j\rangle \\
&+ \sum_{i\in b}\sum_{j\in a} e^{i((\varphi^a_j-\varphi^b_i)\mathbf{k}_\parallel\cdot\mathbf{x}_\parallel-k_z D)}\langle a^\dagger_i b_j\rangle)] \ ,
\end{aligned}
\tag{C4}
$$

where $w(\mathbf{k})$ are Wannier functions in the momentum space (that we considered identical for the two rings), $\mathbf{k}_\parallel\cdot\mathbf{x}_\parallel \doteq k_x(x_i - x_j) + k_y(y_i - y_j)$, $x_i = \cos\phi_i$, $y_i = \sin\phi_i$ fix the positions of the ring wells in the three dimensional space, $\phi_i = 2\pi i/N$ being lattice sites along the rings. Then we transform annihilation and creation operators to the momentum space $a_i = 1/\sqrt{N}\sum_q e^{i\phi_i q} a_q$ and $b_i = 1/\sqrt{N}\sum_q e^{i\phi_i q} b_q$. We also take into account that $\varphi^a_i = 2\pi i\Phi_a/N$ and $\varphi^b_i = 2\pi i \Phi_b/N$ for $i = 0, .., N-1$. Finally, we get (Eq.7)

$$
\begin{aligned}
\rho(\mathbf{k}) = \frac{|w(k_x,k_y,k_z)|^2}{N}\sum_{i=0}^{N-1}\sum_{j=0}^{N-1}\sum_{q\in\{2\pi n/N\}} \\
\Big[\cos\left[\mathbf{k}_\parallel\cdot\mathbf{x}_\parallel + (q+\frac{\Phi_a}{N})(\phi_i-\phi_j)\right]\langle a^\dagger_q a_q\rangle + \\
\cos\left[\mathbf{k}_\parallel\cdot\mathbf{x}_\parallel + (q+\frac{\Phi_b}{N})(\phi_i-\phi_j)\right]\langle b^\dagger_q b_q\rangle + \\
2\cos\left[\mathbf{k}_\parallel\cdot\mathbf{x}_\parallel + k_z D + (q+\frac{\Phi_a}{N})\phi_i - (q+\frac{\Phi_b}{N})\phi_j\right]\langle a^\dagger_q b_q\rangle\Big] \ .
\end{aligned}
\tag{C5}
$$

1. Expectation values for $U = 0$

In the following, we provide the details of the calculations of the expectation values entering the Eq.(C5), for $U = 0$. The Hamiltonian in the Fourier space reads

$$
H_{BH} = \sum_k[-2t\cos\tilde{k}_a a^\dagger_k a_k - 2t\cos\tilde{k}_b b^\dagger_k b_k - g(a^\dagger_k b_k + b^\dagger_k a_k)]
\tag{C6}
$$

We perform a Bogolubov rotation

$$
\begin{aligned}
a_k &= \sin\theta_k\alpha_k + \cos\theta_k\beta_k \\
b_k &= \cos\theta_k\alpha_k - \sin\theta_k\beta_k
\end{aligned}
\tag{C7}
$$

The Hamiltonian Eq.(C6) can be diagonalized choosing $\tan 2\theta_k = g/t(\cos\tilde{k}_a + \cos\tilde{k}_b)$:

$$
\begin{aligned}
H_{BH} &= \sum_k[\varepsilon_\alpha(k)\alpha^\dagger_k\alpha_k + \varepsilon_\beta(k)\beta^\dagger_k\beta_k] \\
\varepsilon_{\alpha,\beta}(k) &= -t(\cos\tilde{k}_a + \cos\tilde{k}_b) \\
&\mp \sqrt{g^2 + t^2(\cos\tilde{k}_a - \cos\tilde{k}_b)^2}
\end{aligned}
\tag{C8}
$$

where $\tilde{k}_a = k + \Phi_a/N, \tilde{k}_b = k + \Phi_b/N$ and $\pm$ corresponds to the α and β respectively.

The correlation functions result

$$
\begin{aligned}
\langle a^\dagger_k a_k\rangle &= \sin^2\theta_k\langle\alpha^\dagger_k\alpha_k\rangle + \cos^2\theta_k\langle\beta^\dagger_k\beta_k\rangle \\
\langle b^\dagger_k b_k\rangle &= \cos^2\theta_k\langle\alpha^\dagger_k\alpha_k\rangle + \sin^2\theta_k\langle\beta^\dagger_k\beta_k\rangle \\
\langle a^\dagger_k b_k\rangle = \langle b^\dagger_k a_k\rangle &= \frac{\sin 2\theta_k}{2}(\langle\alpha^\dagger_k\alpha_k\rangle - \langle\beta^\dagger_k\beta_k\rangle)
\end{aligned}
\tag{C9}
$$

where $\langle\alpha_k^\dagger\alpha_k\rangle$ and $\langle\beta_k^\dagger\beta_k\rangle$ are given by the usual Bose-Einstein distribution:

$$\langle\alpha_k^\dagger\alpha_k\rangle = \frac{1}{e^{(\varepsilon_\alpha(k)-\mu_\alpha)/k_BT}-1} \qquad \text{(C10)}$$
$$\langle\beta_k^\dagger\beta_k\rangle = \frac{1}{e^{(\varepsilon_\beta(k)-\mu_\beta)/k_BT}-1}$$

where $\mu_{\alpha,\beta}$ are the chemical potentials of the condensates of quasiparticles, k_B is a Boltzmann constant and T is e temperature of the condensate.

The chemical potentials can be obtained by fixing the average number of boson per site (filling). It is convenient to introduce the new variables $\mu = (\mu_\alpha + \mu_\beta)/2$, $\delta = (\mu_\alpha - \mu_\beta)/2$. The partition function of the system is given by

$$Z = \prod_k [1 - e^{-\beta(\varepsilon_\alpha(k)-\mu)}][1 - e^{-\beta(\varepsilon_\beta(k)-\mu)}] \qquad \text{(C11)}$$

where $\beta = 1/k_BT$. The free energy of the system can be calculated from the partition function

$$F = -\frac{1}{N\beta}\ln Z \qquad \text{(C12)}$$

Then the chemical potentials can be fixed solving the following equations:

$$N_\alpha + N_\beta = -\frac{\partial F}{\partial\delta}, \;\; N_\alpha - N_\beta = -\frac{\partial F}{\partial\mu} \qquad \text{(C13)}$$

where the $N_{\alpha,\beta}$ are the numbers of the quasiparticles of the type α and β respectively.It is easy to show, that $N_\alpha+N_\beta = N_T$,where N_T is a total number of the bosonic particles in the system.

---

[1] Pasienski, M. & DeMarco, B. A. High-accuracy algorithm for designing arbitrary holographic atom traps. Optics Express, **16**, 2176 (2008).

[2] Gaunt, A. L. & Hadzibabic, Z. Robust digital holography for ultracold atom trapping. Nature Scientific Rep. **2**, 721 (2012).

[3] Schulz, H.J. & Shastry, B.S. A new class of exactly solvable interacting fermion models in one dimension. Phys. Rev. Lett. **80**, 1924 (1998); Osterloh, A., Amico, L. & Eckern, U. Exact solution of generalized SchulzShastry type models. Nucl. Phys. B **588**, 531 (2000).

[4] Fazio, R. & Van der Zant, H. Quantum phase transitions and vortex dynamics in superconducting networks. Phys. Rep. **355 (4)**, 235 (2001).

[5] Rastelli, G., Pop, I. M., & Hekking, F. W. J. Quantum phase slips in Josephson junction rings. Phys. Rev.B **87**, 174513 (2013).

[6] Li, T. C., Kelkar, H., Medellin, D. & Raizen, M.G. Real-time control of the periodicity of a standing wave: an optical accordion. Optics Express **16**, 5468 (2008).

[7] Amico, L., Osterloh, A. & Cataliotti, F. Quantum many particle systems in ring-shaped optical lattices. Phys. Rev. Lett. **95**, 063201 (2005).

[8] Franke-Arnold, S. et al. Optical ferris wheel for ultracold atoms. Optics Express, **15**, 8619 (2007).

[9] Aghamalyan D., Amico L., and Kwek L.-C., Phys. Rev. A **88**, 063627 (2013).

[10] Raghavan, S., Smerzi, A., Fantoni, S. & Shenoy, S. R. Coherent oscillations between two weakly coupled Bose-Einstein condensates: Josephson effects, π oscillations, and macroscopic quantum self-trapping. Phys. Rev. A **59**, 620 (1999).

[11] Andersen, J. D. & Kenkre, V. M. Self-trapping and time evolution in some spatially extended quantum nonlinear systems: exact solutions. Phys. Rev. B **47**, 11134 (1993).

[12] Abramowitz, M. & Stegun, I. A. *Handbook of Mathematical Functions*. Dover, New York, 1970.
;(1998),
